# To the question of the possibility of influence of the hepatitis C virus on the development of neuropsychological disorders

**DOI:** 10.1192/j.eurpsy.2021.1275

**Published:** 2021-08-13

**Authors:** M. Artemieva, B. Tsygankov, G. Ivanova

**Affiliations:** 1 Psychiatry And Medical Psychology, RUDN University, Moscow, Russian Federation; 2 Psychiatry, Narcology And Psychotherapy, Moscow State University of Medicine and Dentistry, Moscow, Russian Federation

**Keywords:** neuropsychological disorders, influence of the hepatitis C virus

## Abstract

**Introduction:**

Hepatitis C virus (HCV) infection produces a chronic systemic disease that induces chronic hepatitis, cirrhosis and hepatocellular carcinoma. Patients with chronic HCV infection may present with a range of extrahepatic symptoms including neuropsychiatric disorders.

**Objectives:**

The aims of this review are to summarize recent literature looking at the associations between psychosocial and neurocognitive factors and HCV, identify the most common neuropsychological disorders and consider the probable mechanisms of mental and cognitive impairment in patients with HCV.

**Methods:**

PubMed/Medline was systematically searched for psychosocial and neurocognitive factors associated with hepatitis C and patient wellbeing. In this review 83 valid articles were analyzed from 1994 to 2018.

**Results:**

According to the literature review in the group of HCV-positive patients were found a significant decrease in higher cognitive functions: memory impairment, concentration and listening. These manifestations of cognitive dysfunction are supposed to be similar to the early symptoms of Alzheimer’s disease. An increased risk of developing dementia has also been noted. The most frequently diagnosed symptoms were fatigue and sleep disturbances, associated with mood disorders diagnosed in 19,2% of cases. Several mechanisms have been considered to explain the pathogenesis of neuropsychiatric disorders observed in chronic HCV infection: 1) the concept of the direct neuroinvasion of HCV; 2) derangement of metabolic pathways; 3) cerebral or systemic inflammation.

**Conclusions:**

To date, the mechanisms of various mental and neurological disorders in patients with chronic HCV infection have been partially identified, but the long-term effect of these changes requires further study.

